# Practical Microwave Synthesis of Carbazole Aldehydes for the Development of DNA-Binding Ligands

**DOI:** 10.3390/molecules24050965

**Published:** 2019-03-09

**Authors:** Agata Głuszyńska, Bernard Juskowiak

**Affiliations:** Laboratory of Bioanalytical Chemistry, Faculty of Chemistry, Adam Mickiewicz University, Umultowska Street 89b, 61-614 Poznań, Poland; juskowia@amu.edu.pl

**Keywords:** microwave synthesis, G-quadruplex ligands, carbazole derivatives, equilibrium dialysis method

## Abstract

Microwave formylation of carbazole derivatives was investigated and 3-monoaldehydes were obtained in high yield. A potential DNA-binding ligand, 3-[(3-ethyl)-2-vinylbenzothiazolium]-9-*N*-ethyl carbazole iodide, was synthesized and characterized including spectral properties (UV-Vis absorption and fluorescence spectra). The binding selectivity and affinity of three carbazole ligands for double-stranded and G-quadruplex DNA structures were studied using a competitive dialysis method in sodium- and potassium-containing buffer solutions.

## 1. Introduction

G-quadruplexes (G4) are nucleic acids structures formed by guanine-rich regions of the human genome. Such G-rich tracts have been found in human telomeres [1] and in promoter regions of certain oncogenes, including *c-MYC* [2], *c-KIT* [3], *bcl-2* [4] or *RET* (REarranged during Transfection) [5]. Moreover, the existence of G4 structures has been proved in human cells [6]. These four-stranded structures are formed by π-π stacking on top of each G-tetrads composed of four Hoogsteen-paired coplanar guanines. They are also stabilized by monovalent cations such as potassium and sodium and can be further stabilized by specific ligands [7,8,9]. A large number of G-quadruplex stabilizing ligands have been described as promising precursors for drug development in anticancer therapy. A large group of ligands has been identified to induce and stabilize G4 formation in telomeric DNA and inhibit telomerase activity in vitro. In addition, a large number of small molecules are potent G-quadruplex ligands that exhibit anti-proliferative activity in cells [10,11,12].

Our research group is interested in the synthesis of ligands containing carbazole skeleton, which can bind to DNA secondary structure of guanine-rich sequence [13,14,15,16]. The designed ligands interact with the G-quadruplex via external stacking due to the presence of a planar carbazole unit and a vinyl benzothiazolium arm coupled with delocalized π-electrons. Carbazole derivatives are very interesting compounds because of their biological and photophysical properties. They are able to target DNA structures, some of them have the potential for development into anticancer drugs [17]. To enhance bioactivity of carbazole derivatives one can introduce other functional groups into carbazole skeleton e.g., imidazole or triazole rings. Imidazole and triazole derivatives, as well as carbazole-based compounds, play an important role in medicinal chemistry because many of them exhibit various biological properties [18,19]. 

The synthesis of aromatic aldehydes is an interesting issue because these compounds are very useful substrates and powerful intermediates for the construction of various natural or bioactive molecules. The Vilsmeier-Haack reaction is one of the methods for the preparation of aldehydes by creating new carbon-carbon bonds. This is a particularly effective method of synthesis of *N*-alkyl-3,6-diformylcarbazoles in high yield by direct Vilsmeier-Haack formylation employing DMF and phosphorus oxychloride used in large (18.8–24.3 equiv) [20,21] or a slight excess (2.5–3 equiv) [22]. However, *N*-alkyl-substituted derivatives as *N*-acetoxyundecyl-3,6-diformyl carbazole has already been obtained with a lower 42% yield [23]. *N*-alkyl-monoformylcarbazole derivatives were also synthesized but using a small excess of formylation reagent (1-6.5 equiv) during 6-8 h with yields of 40%–79% depending on the applied procedure [20,21,24,25,26]. 

Herein, we present the realization of microwave-based approach for monoaldehyde synthesis that has resulted in improvement of the synthesis route (short time, flexibility) and permitted obtaining a high yield of synthesis. In this paper, we also describe the spectral characteristics of one of the G-quadruplex ligand (**3c**) and compare the selectivity of ligands **3a**–**3c** to double-stranded and G-quadruplex structures using the technique of equilibrium dialysis. 

## 2. Results and Discussion

### 2.1. Chemistry

**Scheme 1:**
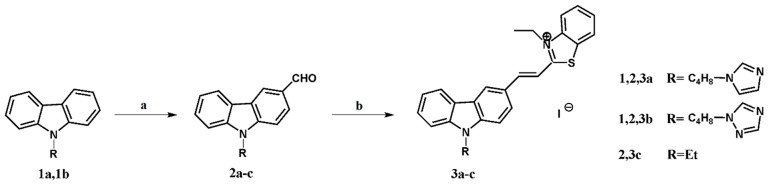
Synthesis of G-quadruplex carbazole ligands. Reagents and conditions: (a) **1a** or **1b**, phosphoryl chloride (POCl_3_, 3 equiv.), *N*,*N*-dimethylformamide (DMF, 3 equiv.), 0 *°*C → 25 *°*C (1 h), microwave (MW) (150 W), 100 *°*C (100 min); (b) **2c**, 3-ethyl-2-methyl-benzothiazolium iodide, piperidine, MeOH, 25 *°*C, 21 h.

The key step in the synthesis of carbazole derivatives **3a**–**3c** suitable as G-quadruplex ligands influencing the reduction of overall yield is the formylation of the aromatic molecule (Scheme 1). The starting carbazole derivatives **1a** and **1b** were prepared from carbazole by known synthetic procedures in 62 and 60 overall yield, respectively [27]. Using nearly stoichiometric amounts of reagents, like Xu and Song [20,21], we obtained monoaldehydes **2a** and **2b** with low 13%–22% yield. The reactions lasted from 6 to 8 h and were assumed as complete when the amount of the product ceased to increase. That is why in our earlier work we have used higher excess of formylating reagents expecting a better yield of mono products but with the simultaneous formation of bis-substituted derivative [13,16]. Therefore, we decided to improve the originally used method, testing microwave-assisted chemistry. The microwave technique offers a lot of advantages, it is fast, the reactions are clean, simpler, efficient and economic [28,29].

In preliminary experiments, 9-(4-(1*H*-imidazol-1-yl)butyl)-9*H*-carbazole **1a**, chosen as a representative substrate, was subjected to the microwave-assisted treatment with Vilsmayer’s formylating reagent under different reaction conditions. Preliminary experiments with 1 and 2 equivalents of formylating reagent showed a rather low conversion, as analyzed by TLC on the crude product, for the presence of starting substrate (Table 1, entries 1 and 2). When carbazole derivative **1a** was treated with 3 equiv. of the reagent, monoaldehyde **2a** was obtained in 82%–88% yield (entries 3 and 4). Increasing the temperature resulted in the faster formation of a bis-substituted product and decreasing yield of the monosubstituted derivative of carbazole (entry 5). A slight increase in the reagent content up to 4 equiv. caused a significant reduction of yield to 58% (entry 6). The use of a large excess of the reagent (19 equiv.) resulted in obtaining monoaldehyde **2a** after only 15 min but in 51% yield (entry 7). In the last two cases (entries 6 and 7), the formation of the bisaldehyde derivative was observed as proven by TLC analysis. In summary, the optimized reaction conditions of Vilsmayer formylation of 9-(4-(1*H*-azol-1-yl)butyl)-9*H*-carbazoles were established as follows: the use of 3 equivalents of formylating reagent at 100 °C for 100 min under microwave irradiation (150 W) (3.2.1). In the optimized reaction conditions, we have also carried out the reaction with derivative **1b** and the result is illustrated in Table 1 (entry 8). 

To demonstrate the usefulness of carbazole monoaldehyde derivatives, ligands **3a**–**3c** were prepared by the Knoevenagel type condensation between monoaldehydes **2a**–**2c** and 3-ethyl-2-methylbenzothiazolium iodide in MeOH. As we have reported recently, synthesis of ligand **3a** was carried out without piperidine (pKa 11.22), because the basic nature of imidazole (pKa 14.5), was sufficient to the progress of the reaction and the experiments with the addition of piperidine proceeded with a lower yield [16,30]. In the case of derivatives **3b** and **3c**, the use of piperidine as a basic catalyst was necessary. In the case of ligand 3b, the basicity of the triazole itself (pKa 10.3) was insufficient for the reaction [13]. In the synthesis of ligand 3c, the basicity of the piperidine as a catalyst was sufficient for synthesis and it was not necessary to use sodium methoxide as a stronger base [31]. Ligands were characterized by the positive modes *m*/*z* 477, 478, 383 [M]^+^ and a negative mode *m*/*z* 127 [M]^−^ peaks in the ES-MS, respectively for ligands **3a**-**3c**, and by ^1^H-NMR spectral data. Their *E* configuration was established on the basis of a high value of the coupling constants *J* = 15.5, 15.4, 15.4 Hz for protons of the CH=CH double bond, found as an isolated doublet at δ 8.03, 8.03, 8.02 and a doublet in the group of aromatic protons at δ 7.77, 7.78, 8.42, respectively for ligands **3a**–**3c**. The purity of crystalline ligands was checked by HPLC with absorption and fluorescence detectors.

### 2.2. Spectral Properties of Ligand ***3c***

**Figure 1:**
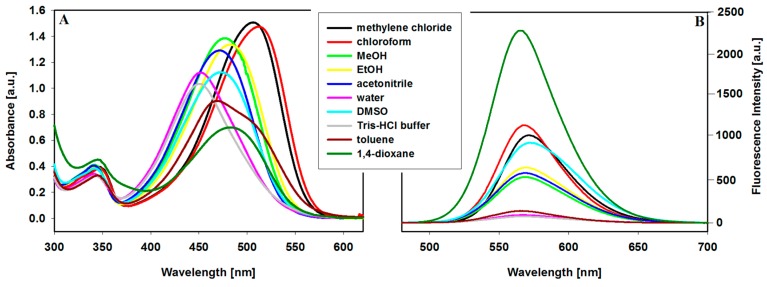
(**A**) Absorption spectra ([**3c**] = 2.9 × 10^−5^ M) and (**B**) fluorescence spectra ([**3c**] = 4.4 × 10^−6^ M) of the dye **3c** in selected organic solvents and aqueous solutions at room temperature (λ_ex_ = Abs. λ_max_).

Carbazole ligands **3a** and **3b** with azole groups have already been characterized concerning spectral properties (UV-Vis absorption spectra, fluorescence spectra) [13,16]. That is why we decided to investigate the effect of different solvents on the UV-Vis and fluorescence properties of ligand **3c**. This carbazole derivative, as well as the two previous ones, is soluble and stable in selected organic solvents and aqueous solutions (Figure 1A). In CH_2_Cl_2_ and CHCl_3_, absorption bands are red-shifted to 504 and 509 nm, respectively, compared with those in other solvents with λ_max_ below 483 nm. The UV-Vis absorption spectra, with a maximum absorption band at about 480 nm characteristic of a double bond carbon-carbon between carbazole and benzothiazolium group are correlated with the polarity of aprotic and protic solvents - large solvatochromism (58 nm) and changes in absorptivity were observed (Figure 1A, Table 2).

The fluorescence spectra of ligand **3c** recorded in different solvents and aqueous solutions are shown in Figure 1B. The compound exhibit emission band, at ca. 570 nm, which intensity is connected with the polarity of the aprotic and protic solvents (Table 2). The intensity of fluorescence decreased with the increasing polarity of the solvent and the lowest was observed in aqueous solutions. The fluorescence band in the spectrum recorded in nonpolar 1,4-dioxane exhibits the highest intensity. On the other hand, in toluene, the second non-polar solvent, fluorescence was quenched and its intensity was on the level of aqueous solutions of ligand **3c**. The same effect was observed for the carbazole ligand with triazole substituent [13]. It seems that the low fluorescence intensity of the ligand is associated with the loss of its flat structure by the rotation of benzothiazolium arm coupled by double bond C=C with a carbazole skeleton [32] or self-quenching due to dimer formation.

It should be noted that we practically did not observe any systematic spectral changes in the three carbazole derivatives.

### 2.3. DNA Binding

The affinity of the interactions of 9-*N*-substituted carbazole derivatives **3a**–**3c** with DNA has been already studied by UV-Vis, fluorescence and CD spectroscopic techniques as well as molecular modeling [13,14,15,16]. It was found that these ligands interacted with G-quadruplexes via external stacking thanks to the presence of a planar carbazole unit and a vinylbenzothiazolium arm coupled with delocalized π-electrons. Among the methods used to evaluate the selectivity and the affinity of the ligands to different DNA structures [33,34,35,36,37], our research group uses mainly UV-Vis spectrophotometry, fluorescence and CD spectroscopy [12,13,14,15]. In our study, we excluded the FRET-based fluorescent methods because we observed fluorescence quenching between the used fluorophores (thiazole orange) and the tested G4-binders (carbazoles) in FID experiments [13]. In this paper, we present results of competitive dialysis experiments carried out using eight nucleic acid structures. Among the methods suitable for investigation of ligand binding to different structures of nucleic acids (single-stranded, double helix, triplex or tetraplex) of different topologies (anti-parallel or parallel quadruplex), the equilibrium/competition dialysis method is particularly powerful. The advantages of this method are many, including its speed and the possibility to study many samples of nucleic acids in the same conditions at the same time. In addition, a study of small molecules that selectively recognize the unique structural features of nucleic acids, is relatively easy [36,37]. In the competition dialysis procedure, equal volumes of chosen DNA samples (the same concentrations) are equilibrated by dialysis with a ligand’s dialysate solution. The experiment is carried out for 24 h, then the amount of the bound ligand is determined by UV or fluorescence spectroscopy. This method can be carried out in various buffers such as Tris-HCl buffer, sodium cacodylate, sodium and potassium phosphate, 4-(2-hydroxyethyl)-1-piperazineethanesulfonic acid (HEPES) and other, as well as using different NaCl or KCl concentrations [36,37]. Mainly, however, various sodium-based buffers were used [34,36,37,38,39,40,41,42,43,44,45]. To investigate the selectivity of ligands for G-quadruplexes formation in the buffer in the presence of potassium specific structures, it seems more reasonable to conduct the experiments under such conditions. In the experiments carried out in sodium-based buffers after the dialysis the SDS (sodium dodecyl sulfate) solution was added, in order to dissociate the ligand from the DNA complex and to perform spectral measurements. But when working with buffers in the presence of potassium ions, the addition of SDS leads to the formation of a white precipitate. Ferreira et al. have solved this problem by digesting the complex (DNA) with snake venom phosphodiesterase at the end of the dialysis experiment [46,47,48]. Alternative way includes the application of other surfactants e.g., nonionic, to dissociate the ligand-DNA complex [36,37]. We decided to check this simpler way by using Triton X-100 to break up the ligand-DNA complex.

**Figure 2:**
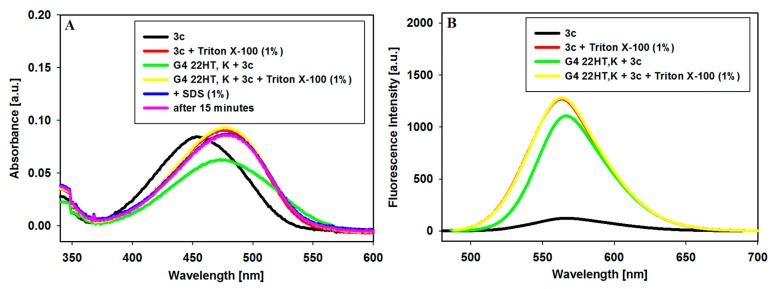
The effect of Triton X-100 on dissociation the ligand **3c** (3 µM) from the DNA complex in potassium-based buffer. (**A**) Absorption spectra and (**B**) fluorescence spectra; conditions: [L] = 3 µM, [22HT G4] = 3 µM, 10 mM Tris-HCl buffer (pH 7.2) containing 100 mM KCl; λ_ex_: 453 and 478 nm.

After addition of Triton X-100 (1% (*w*/*v*)) to solution of ligand **3c** its absorption maximum at 453 nm on the UV-Vis spectrum, characteristic of carbazole chromophore conjugated with benzothiazolim system via double bond C=C, was red-shifted to 478 nm (Δλ = 25 nm) and a slight hyperchromicity (6.6%) was observed (Figure 2A). When Triton X-100 was added to the 22HT G4 complex with the same amount of the ligand in KCl-based buffer, the ligand was released from the complex and there was no clouding of the solution. Further, the SDS addition (1% (*w*/*v*)) did not change the spectrum, nor was it observed after 15 min (Figure 2A). The spectra of the free ligand and the ligand released from the complex in the presence of Triton X-100 practically overlapped. After the addition of Triton X-100 to a solution of ligand **3c**, its emission increased significantly (Figure 2B). When Triton X-100 was added to the 22HT G4 complex, the ligand was released from the complex and its spectrum overlapped with the emission spectrum of the free ligand in the presence of Triton X-100 (Figure 2B). As a result, we were able to use Triton X-100 in an equilibrium dialysis experiment to investigate the selectivity of carbazole ligands to G-quadruplexes with sequences related to different proto-oncogenes in a buffer containing potassium ions.

**Figure 3:**
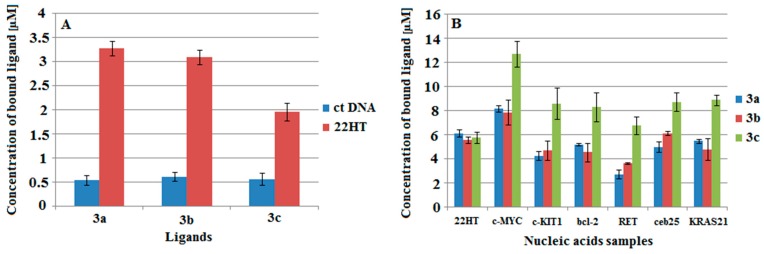
Results of competition dialysis experiment (**A**,**B**). The amount of carbazole ligands bound to the ctDNA and various G-quadruplexes is shown as a bar graph. The free ligand concentration in the experiment was 1 µM, and the total concentration of each G-quadruplex was 75 µM per G4 as a monomer unit. Structures of G-quadruplexes are described in Section 3.2 and Table 3.

Preliminary studies on the DNA binding affinity of the ligands were carried out in a sodium ion-containing buffer with calf thymus DNA as a double-stranded DNA (dsDNA) and 22-mer oligonucleotide (5′-AGGGTTAGGGTTAGGGTTAGG G-3′), with a sequence related to human telomere DNA (G4 DNA), which can form intramolecular G-quadruplex structure by folding of a single oligonucleotide molecule. Very weak interactions with the double-stranded structure were observed for all ligands. A much higher affinity of ligands to the four-stranded structure was noticed (Figure 3A).

The affinity of ligands to 7 different forms of nucleic acids was also studied in the potassium-based buffer at room temperature. The samples of DNA included structures of G-quadruplexes represented by oligonucleotides which had human telomere sequence (22HT) and human protooncogene sequences (*c-MYC*, *c-KIT1*, *bcl-2*, *RET*, *ceb25*, *KRAS21*) (Table 3). All can form intramolecular G-quadruplex structures by folding of a single oligonucleotide molecule. 

After the dialysis equilibrium experiment, the ligand concentration in each sample was determined by the fluorescence method. Bound ligand concentration was determined using the fluorescence calibration curve. Fluorescence spectra were taken immediately after the addition of SDS or Triton X-100 solution in order to dissociate the ligand from the DNA complex, respectively in sodium- or potassium-based buffer. In order to show results of equilibrium dialysis experiments in a more distinct way, the amounts of bound ligands to each DNA were shown as bars in Figure 3B.

Results indicate that tested ligands have a high affinity for all tetraplexes, with a remarkable binding preference for c-MYC G-quadruplex. The changes observed in these experiments were stronger for ligand **3c**, and such differences in the interaction were not observed during the classical spectrophotometric and fluorescence titration experiments. However, the trend of binding affinity of the other two ligands **3a** and **3b** with the azole substituents is consistent with previous experiments: *c-MYC* > *KRAS21* ~ *bcl-2* ~ 22HT/K > *c-KIT1* > 22HT/Na [14,15,16].

## 3. Materials and Methods

### 3.1. General Information

Melting points: determined on a Koffler block and not corrected. IR spectra: FT BRUKER IFS 66 v/S in KBr pellets, (Karlsruhe, Germany). ^1^H- and ^13^C-NMR spectra: Varian Gemini 400 (Oxford, UK) with TMS as an internal standard. Mass spectra (ESI): Waters & Micromass mass spectrometer ZQ (Manchester, UK). Analytical HPLC: Waters HPLC system 1525 equipped with a Photodiode Array detector 2998 and a fluorescence detector 2475 (Waters Corporation, Milford, MA, USA), a Breeze interface. A Symmetry^®^ C18 column (d_p_ = 3.5 μm, 75 × 4.6 mm) (Waters) was used with isocratic elution (70% MeOH and 30% 10 mM NaCl, flow rate 1 mL min^−1^). Column chromatography was carried out with silica gel grade 60, 0.063–0.200 mm (70–230 mesh). Microwave reactions were performed in the MARS 6 reactor (CEM Corporation, NC, USA). The microplate reader (Infinite M200 Tecan, Männedorf, Switzerland) and the Slide-A-Lyzer MINI Dialysis Units (Pierce, Rockford, IL, USA) were used in the equilibrium dialysis method.

### 3.2. Materials

9-Ethyl-3-formylcarbazole (CAS Number 7570-45-8), Tris Base (CAS Number 77-86-1), Tris HCl (CAS Number 1185-53-1), Triton X-100 (CAS Number 9002-93-1) and SDS (CAS Number 151-21-3) were obtained from Aldrich Chemical Co. (Poznań, Poland) and used as received. All commercial reagents and solvents for synthesis were obtained from Aldrich Chemical Co. and used without purification, unless otherwise reported. A Milli-Q filtered water (Millipore Co., Burlington, MA, USA) was used throughout.

Double-stranded DNA (ctDNA) was purchased from Sigma-Aldrich (CAS Number 73049-39-5). G-quadruplexes were represented by oligonucleotides which had human telomere sequence 5′-AGGG(TTAGGG)_3_-3′ (22HT) and human oncogene sequences 5′-TGAGGGTGGGTAGGGTGGGTAA-3′ (*c-MYC*), 5′-AGGGAGGGCGCTGGGAGGAGGG-3′ (*c-KIT1*), 5′-GGGCGCGGGAGGAATTGGGCGGG-3′ (*bcl-2*), 5′-GGGGCGGGGCGGGGCGGGGT-3′ (RET), 5′-AAGGGTGGGTGTAAGTGTGGGTGGGT-3′ (*ceb25*), 5′-AGGGCGGTGTGGG AAGAGGGA-3′ (*KRAS21*) (all can form intramolecular G-quadruplex structures by folding of a single oligonucleotide molecule). Synthetic oligonucleotides were purchased from Genomed (Poland) and were used without further purification. The strands concentrations were determined at 260 nm at 85 °C using extinction coefficients of 251,800 M^−1^ cm^−1^ (22HT), 257,600 M^−1^ cm^−1^ (*c-KIT1*), 254,600 M^−1^ cm^−1^ (*c-MYC*), 258,300 M^−1^ cm^−1^ (bcl-2), 214,900 M^−1^ cm^−1^ (*RET*), 295,000 M^−1^ cm^−1^ (*ceb25*), 251,300 M^−1^ cm^−1^ (*KRAS21*) as calculated from the published values of molar absorptivities of nucleotides [49].

#### 3.2.1. General Procedure of Synthesis of Compounds **2a** and **2b**

To *N*,*N*-dimethylformamide (0.231 mL, 3 mmol, 3 equiv) purged with argon and cooled at 0 °C, phosphoryl chloride (0.279 mL, 3 mmol, 3 equiv) was added dropwise. The mixture was then slowly heated to room temperature (rt) and stirred for 1 h. The carbazole derivative **1a** or **1b** (1 mmol) dissolved in DMF (0.3 mL) was added dropwise. Then the reaction was carried out in a microwave reactor with a power 150 W at 100 °C. After 1 h, 0.3 mL DMF was added. When no more starting material was present in the reaction mixture (TLC, 100 min), it was cooled to rt and poured into ice. After rt was reached the phases were separated and the aqueous one was extracted with chloroform/2-propanol (3:1) three times (30 mL each). The aqueous solution was neutralized by 25% NaOH and then extracted with chloroform/2-propanol (3:1) three times (30 mL each). The organic phase was washed with water and brine. After the standard work-up of organic phase, the crude product **2a** or **2b** were purified by column chromatography (crude product **2a**: silica gel, 1:20 methanol:dichloromethane = 1.2%) to give monoaldehyde derivative 2a in 88 % and crude product **2b**: silica gel, 1:15 hexane:dichloromethane = 2:3 to give monoaldehyde 2b in 86 %. The structure of the compounds was confirmed by spectroscopy and was consistent with the early published data [13,16].

#### 3.2.2. Synthesis of 3-[(3-ethyl)-2-vinylbenzothiazolium]-9-*N*-ethyl carbazole iodide **3c**

To a stirred orange solution of 9-ethyl-3-formylcarbazole **2c** (446.6 mg, 2 mM) and 3-ethyl-2-methyl-benzothiazolium iodide (625.6 mg, 2.05 mM) in MeOH (10 mL) piperidine (0.159 mL, 1.6 mM) was added. The mixture was stirred at this temperature for 21 h. The solid was filtered off, washed with hexane and dried to give 897.6 mg (Y: 88 %) of compound **3c**. The purity of ligand was examined by the HPLC technique with absorption and fluorescence detectors. *Trans*-ligand was used without further purification.

M.p.: 252–254 °C. IR (KBr) cm^−1^: 1644 (C=N), 961 (E CH=CH). ^1^H-NMR (DMSO-*d_6_*) δ: 1.37 (t, *J* = 7.1 Hz, 3H, N-CH_2_-C**H**_3_), 1. 51 (t, *J* = 7.1 Hz, 3H, N^+^CH_2_-C**H**_3_), 4.53 (q, *J* = 7.1 Hz, 2H, N-C**H**_2_-CH_3_), 4.99 (q, *J* = 7.1 Hz, 2H, N^+^C**H**_2_-CH_3_), 7.35 (t, *J* = 7.5 Hz, 1H, ArH), 7.56 (t, *J* = 7.3, 1H, ArH), 7.71–7.79 (m, 1H, ArH), 7.77 (d, *J* = 15.5 Hz, 1H, CH=C**H**), 7.82–7.88 (m, 2H, ArH), 8.03 (d, *J* = 15.5 Hz, 1H, C**H**=CH), 8.25–8.27 (m, 3H, ArH), 8.41–8.45 (m, 2H, ArH), 8.94 (s, 1H, ArH). ^13^C-NMR (100 MHz, DMSO-*d_6_*): δ (ppm) 171.5, 151.2, 142.2, 140.9, 140.3, 129.3, 128.2, 128.0, 127.8, 126.8, 125.1, 124.3, 124.0, 122.9, 122.2, 120.7, 120.3, 116.3, 110.1, 109.4, 44.2, 37.4, 14.2, 13.8. ESI-MS: (positive mode) *m/z* 383 [M^+^], (negative mode) 127 [M^−^].

### 3.3. Methods

#### 3.3.1. Absorption and Fluorescence Spectroscopy 

The absorption spectra were recorded on a Cecil CE-2021 spectrophotometer in the 200–700 nm range at 25 °C. The fluorescence measurements were carried out using a Jasco FP 8200 spectrofluorimeter. The cell compartments were thermostated at 25 °C. The fluorescence spectra were collected from 510 to 750 nm with both excitation and emission slits being 5 nm. All of the measurements were carried out using a 10 mm quartz cell.

#### 3.3.2. Dialysis Assay Method

Nucleic acids samples were prepared at the same concentration of 75 µM in terms of the monomeric units. G-quadruplexes were initially heated at 95 °C for 5 min in a block heater followed by slow cooling to room temperature (4 h). All samples were incubated at 4 °C for 24 h prior to the dialysis experiment. The Slide-A-Lyzer MINI Dialysis Units (Pierce, Rockford, IL) were soaked in 1L of distilled water for 15 min. The dialysis units (MWCO-molecular weight cut off: 3500), each containing a 125 µL volume of different nucleic acid and one blank unit (buffer only), were put into a beaker filled with a dialysate solution—1µM ligand solution in Tris HCl buffer (10 mM, pH=7.2) containing 105 mM NaCl or KCl. The beaker was covered with parafilm and aluminium foil and equilibrated with continuous stirring for 24 h at room temperature. At the end, 100 µL of each nucleic acid sample was transferred to a 96-well microplate (fluorescent plates Cat# 3993, Corning Inc., Corning, NY, USA) and SDS (sodium dodecyl sulfate) or Triton X-100 solution were added to dissociate the ligand from the DNA complex, respectively in sodium or potassium buffers (a final concentration of 1% (*w*/*v*) was used). Emission spectra of all samples were recorded with a microplate reader (Infinite M200 Tecan, Austria). The concentration of bound ligand (C_b_) was calculated by difference as C_b_ = C_t_ − C_f_, where the total ligand (C_t_) and free ligand (C_f_) concentrations were determined using the fluorescence calibration graphs. Free ligand concentration (reagent blank) did not vary appreciably from the initial value of 1 µM.

## 4. Conclusions

In conclusion, a new synthesis procedure was developed to obtain carbazole monoaldehydes in high yield using microwave irradiation. The usefulness of carbazole monoaldehyde derivatives was demonstrated by the preparation of ligands **3a**–**3c**. The spectral properties of *N*-ethyl derivative **3c** were characterized and the ligand could stabilize G-quadruplex formed from c-MYC and c-KIT1 gene promoter region. To evaluate the selectivity and the affinity of carbazole ligands for double-stranded and G-quadruplex DNA structures we used a competitive dialysis method. The experiments were conducted in sodium- or potassium-based buffer, and to dissociate the ligand from the DNA complex, SDS or Triton X-100 were added respectively. Very weak interactions with the double-stranded structure were observed for all ligands. Results of experiments carried out in potassium-based buffer indicate that tested ligands have high affinity for all tetraplexes, with a remarkable binding preference for c-MYC G-quadruplex.

## Figures and Tables

**Table 1:** Optimization of microwave-assisted Vilsmayer’s formylation.

Entry	Substrate	Vilsmayer Reagent [equiv.]	T [°C]	t [min] ^a^	Y [%] ^b^
1	1a	1	100	120	49
2	1a	2	100	120	58
3	1a	3	100	80	82
4	1a	3	100	100	88
5	1a	3	120	60	78
6	1a	4	100	90	58
7	1a	19	100	15	51
8	1b	3	100	100	86

^a^ Monitored using TLC. ^b^ Isolated yield.

**Table 2:** Effect of solvent on the spectral parameters in the absorption and fluorescence spectra of ligand **3c** (mean values ± SD of three determinations).

Solvent	ε/10^4^ [M^−1^∙cm^−1^]	Abs. λ_max_ [nm]	Em. λ_max_ [nm]	Stockes Shift [nm]	Relative Fluorescence Yield [%]
CH_2_Cl_2_	5.6 ± 0.1	504	571	67	45.5
CHCl_3_	5.5 ± 0.1	509	567	58	49.9
MeOH	5.2 ± 0.1	475	568	93	22.2
EtOH	5.1 ± 0.1	480	570	90	26.2
ACN	4.8 ± 0.1	470	569	99	22.3
H_2_O	4.3 ± 0.1	452	568	116	3.2
DMSO	4.2 ± 0.1	472	572	100	31.2
10 mM Tris-HCl	3.9 ± 0.1	451	567	116	2.3
Toluene	3.3 ± 0.1	468	567	99	3.7
1,4-Dioxane	2.4 ± 0.1	483	565	82	42.9

**Table 3:** Nucleic acids samples used in equilibrium dialysis assay.

Structure	Sample	DNA or Oligonucleotide	Monomer Unit
Double-stranded	ctDNA	Calf thymus DNA	Base pair
Tetra-stranded	22HT	5′-AGGGTTAGGGTTAGGGTTAGGG-3′	quadruplex
	*c-MYC*	5′-TGAGGGTGGGTAGGGTGGGTAA-3′	
	*c-KIT1*	5′-AGGGAGGGCGCTGGGAGGAGGG-3′	
	*Bcl-2*	5′-GGGCGCGGGAGGAATTGGGCGGG-3′	
	*RET*	5′-GGGGCGGGGCGGGGCGGGGT-3′	
	ceb25	5′-AAGGGTGGGTGTAAGTGTGGGTGGGT-3′	
	*KRAS21*	5′-AGGGCGGTGTGGGAAGAGGGA-3′	
